# Circulating proteins and risk of small vessel stroke: A two-sample Mendelian randomization study

**DOI:** 10.1097/MD.0000000000048898

**Published:** 2026-05-22

**Authors:** Ziwei Gao, Yawen Xu, Yue Chen, Dezhi Kang, Yuanxiang Lin, Peisen Yao

**Affiliations:** a Department of Neurosurgery, Neurosurgery Research Institute, The First Affiliated Hospital, Fujian Medical University, Fuzhou, Fujian, China; b Department of Neurosurgery, National Regional Medical Center, Binhai Campus of the First Affiliated Hospital, Fujian Medical University, Fuzhou, Fujian, China; c Fujian Provincial Institutes of Brain Disorders and Brain Sciences, First Affiliated Hospital, Fujian Medical University, Fuzhou, Fujian, China; d Fujian Provincial Clinical Research Center for Neurological Diseases, First Affiliated Hospital, Fujian Medical University, Fuzhou, Fujian, China.

**Keywords:** causal effect, circulating proteins, GRAMD1C, Mendelian randomization, PTGR1, small vessel stroke

## Abstract

To identify promising circulating biomarkers for small vessel stroke (SVS), we applied Mendelian randomization (MR) design to systematically screen circulating proteins for potential development of SVS. We performed two-sample MR analyses to estimate the associations between 4782 human circulating proteins (including immunity-related proteins) and risk of SVS. We selected 359 circulating proteins in MR estimation (4422 circulating proteins with <3 SNPs were excluded) with totally 5368 European descents. Summary statistics for SVS originated from the MEGASTROKE Consortium, involving 446,696 participants. MR analyses were conducted to estimate associations of circulating proteins with SVS. After inverse variance weighting and sensitivity analysis filtration, GRAMD1C causally increased the risk of SVS (OR = 2.86, 95% CI = 1.71–4.76, *P* = 5.61e−05). In addition, PTGR1 (OR = 1.15, 95% CI = 1.04–1.27, *P* = 6.65e−03) presented a suggestive association with SVS. In this two-sample MR investigation, a significant association was identified between the cholesterol transporter GRAMD1C and SVS, indicating its potential as a promising target for diagnosis and therapy.

## 1. Introduction

Approximately 25% of strokes are classified as small vessel strokes (SVS).^[1]^ Around 9.6% of individuals with SVS will have a stroke recurrence within a 3-year period. Responsible for vascular cognitive impairment, this condition is the primary cause of dementias, movement disorders, Parkinson disease, balance problems, falls, and psychiatric symptoms such as depression, apathy, and personality changes. The impact is significant on families and society in low- and middle-income countries.^[2,3]^ SVS is commonly associated with arteriosclerosis caused by aging, hypertension, and other typical vascular risk factors, while amyloidosis vascular disease is also a common cause in older individuals.^[4]^

The clinical manifestations of SVS are varied, with the onset being either urgent or gradual and lacking specificity.^[4]^ Because of the small size of the lesion, many patients experience either no symptoms or only mild symptoms. The clinical manifestations and course of SVS can vary greatly due to differences in location, type, and severity of the lesion, as well as individual brain reserve, other elastic factors, and comorbidities.^[5]^ The diagnosis of SVS depends on imaging examinations, with magnetic resonance imaging being the most crucial tool for detecting features such as recent small subcortical infarcts, lacunars, white matter hyperintensities, perivascular spaces, microbleeds, and micro infarcts.^[6]^ However, magnetic resonance imaging is expensive, time-consuming, and is less commonly performed in daily physical examinations. The screening of SVS requires more noninvasive and convenient methods.

Nevertheless, the genetic factors influencing widespread circulating proteins in the progression of SVS have been disregarded. Although large-artery stroke shares common vascular risk factors, compelling evidence from genetic studies indicates that these stroke subtypes have largely distinct genetic architectures. A combined analysis of all stroke subtypes could obscure signals unique to SVS due to etiological heterogeneity. Therefore, we focused specifically on SVS to uncover causal pathways that are most relevant to SVS. Mendelian randomization (MR) serves as a robust statistical tool for evaluating causal links between exposures and outcomes.^[7,8]^ In this study, we employed a two-sample MR analysis to assess the causal effects of 4782 human circulating proteins, which also include immunity-related proteins, on SVS as identified by the MEGASTROKE consortium.

## 2. Methods

### 2.1. Study design

**Figure 1.**
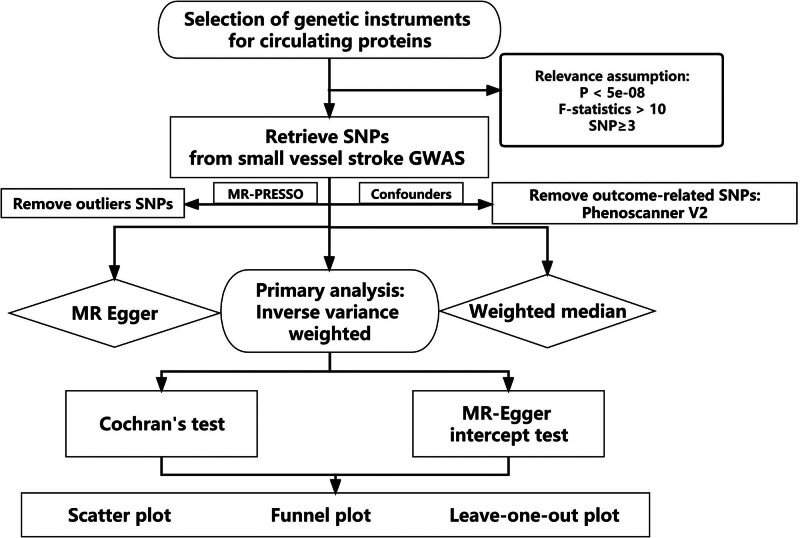
Overview of the current Mendelian randomization (MR) study. GWASs = genome-wide association studies.

We conducted a thorough evaluation of the potential causal link between 4782 circulating human proteins and the likelihood of SVS through two-sample MR analyses. A convincing MR design should be in compliance with 3 fundamental assumptions: genetic instruments are robustly associated with 4782 human circulating proteins; genetic instruments are not associated with confounders; and genetic instruments influence the SVS only through exposures of interest.^[9]^ The independence of horizontal pleiotropy, which includes the second and third assumptions, can be tested using a range of statistical methods.^[10]^ Genetic information for SVS was obtained from the MEGASTROKE consortium (https://www.megastroke.org/download.html), a large-scale international collaboration launched by the International Stroke Genetics Consortium. The study overview is presented in Figure 1.

### 2.2. GWAS summary datasets of circulating proteins

Four thousand, seven hundred eighty-two human circulating proteins were selected from genome-wide association studies, with a total of 5368 European descendants.^[11]^ The genetic variants analyzed in this research were sourced from genome-wide association scans, along with a comprehensive proteogenomic study carried out by Alexander et al.^[12]^ A total of 5368 European descents were included, and approximately 4035 SNPs were tested.^[11]^ Genetic information for each circulating protein was obtained from the NHGRI-EBI Catalog of human genome-wide association studies (https://www.ebi.ac.uk/gwas/, GCST90086175 to GCST90090956). A total of 359 circulating proteins with more than 3 association loci per circulating protein were identified (Table S1).

### 2.3. Small vessel stroke

SVS GWAS results were recently provided by the MEGASTROKE consortium. Genetic links to SVS were discovered by the European group in the study. This study involved 40,585 cases and 406,111 controls. SVS was determined using clinical criteria as outlined by the World Health Organization and Acute Stroke Treatment (TOAST) criteria.^[13]^

### 2.4. Selection of genetic instruments

We employed multiple genetic variants of 359 circulating proteins as genetic instruments to establish the causal relationships between these proteins and the risk of SVS. The following selection criteria were applied in MR estimates: we used the genome-wide significance threshold <5 × 10^−8^ to obtain top independent SNPs, and we performed a clumping test to assess the linkage disequilibrium (*r*2 threshold of 0.001) with the index variant within a 1 Mb window. Meanwhile, to avoid bias owing to the employment of weak instruments, F statistics were calculated for each SNP to measure the statistical strength as previously described.^[14]^ SNPs with F < 10 were recognized as weak instruments and were discarded to ensure all the SNPs conferred sufficient variance for corresponding metabolites.^[14]^ Finally, circulating proteins with <3 SNPs were excluded from MR analysis.^[15]^ We employed MR pleiotropy residual sum and outlier in order to identify horizontal pleiotropic outliers and to retest following the removal of the outliers.^[16]^

### 2.5. MR analyses

To estimate the influence of educational attainment on each phenotype, we utilized 3 different MR methods: random-effects inverse variance weighting (IVW), the weighted median estimator, and MR-Egger regression. In the principal MR analysis, the IVW method was employed, with MR-Egger and weighted median used to improve the IVW estimates. MR-Egger regression necessitates that the impact of genetic variants on educational attainment remains unaffected by their pleiotropic effects on the outcome.^[17]^ The weighted median MR approach allows for the utilization of invalid instruments as long as over half of the instruments used in the MR analysis are valid. In the IVW analysis, the weighted regression of the SNP-to-exposure effects on the SNP-to-outcome effects is used to calculate the estimate. To decrease the standard error, we imposed a constraint of zero on the intercept in IVW estimation. For significant estimates of IVW, consistent estimates of 3 MR estimates were selected. The intercept test of the MR-Egger identifies horizontal pleiotropy if the intercept from the MR-Egger analysis is not equal to zero. Heterogeneity was enumerated using Cochran Q test.

Additionally, a sensitivity analysis was conducted by leaving out one data point at a time, along with a funnel plot analysis, to eliminate any potential bias. Any results showing significant bias were excluded.

### 2.6. Confounding analysis

Although an array of statistical methods were conducted in sensitivity analysis to evaluate any violation of the MR assumptions, we scanned with the Phenoscanner V2 website (http://www.phenoscanner.medschl.cam.ac.uk/) to explore whether the metabolites-associated SNPs were meanwhile associated with several common risk factors that might bias the MR estimates, including ischemic cardiomyopathy, arterial embolism and thrombosis, and coronary artery disease. SNPs associated with potential confounders were removed, and IVW estimation was re-performed.^[18]^

### 2.7. Steiger filtering on inference direction from exposure to outcome

In order to mitigate the possible negative impacts of reverse association, where the protein is seen as the result and the phenotype as the cause, we employed the Steiger filtering method to determine the accurate directions of inference.^[19]^ We only included the protein-phenotype pairs that demonstrated the accurate direction. The analysis was carried out using the directionality_test function from the R package TwoSampleMR.

### 2.8. Ethics

Since the original GWAS previously received ethics and institutional review board approval, no further ethical approval was obtained in the study.

### 2.9. Statistics

All analyses were performed in R version 4.2.1 using TwoSampleMR (version 0.5.6). The level of statistical significance was established at a two-tailed *P* value of <.05. For outcome-level analyses, given the 359 estimates, a Bonferroni-corrected *P*-value was set as .05/359 (1.39e−04). A Bonferroni-corrected *P*-value of .05/359 (1.39e−04) was deemed significant, while a *P*-value between .05/359 (1.39e−04) and .05 was considered to suggest an association.

## 3. Results

Through the instrument selection steps, 359 circulating proteins were identified for MR estimation. The number of SNPs linked to each circulating protein ranged from 3 to 37, with all F statistics for the SNPs exceeding 10, indicating the absence of weak instruments.

After IVW and sensitivity analysis filtration, GRAMD1C causally increased the risk of SVS (OR = 2.86, 95% CI = 1.71–4.76, *P* = 5.61e−05). The causal direction of GRAMD1C on SVS is confirmed by Steiger filtering (*P* = 1.69e−271). In addition, PTGR1 (OR = 1.15, 95% CI = 1.04–1.27, *P* = 6.65e−03) presented a suggestive association with SVS (Table 1). The causal direction of PTGR1 on SVS is confirmed by Steiger filtering (*P* = 1.22e−121). Cochran Q-derived *P* values indicated that no heterogeneity was detected (Table 1). Besides, intercept terms from MR-Egger suggested a low risk of horizontal pleiotropy (Table 1). All *P* values (IVW estimates) of 359 circulating proteins on SVS are shown in Table S1.

**Table 1:** Significant Mendelian randomization estimates from circulating proteins on small vessel stroke dependence.

Circulating proteins	Methods	OR (95% confidence intervals)	*P* value	Cochran Q-derived *P* value	MR-Egger intercept-derived *P* value
GRAMD1C (GCST90090136)	MR Egger	4.25 (1.32–13.69)	2.13E−02		
	Weighted median	2.26 (1.07–4.77)	3.19E−02		
	Inverse variance weighted	2.86 (1.71–4.76)	5.61E−05	6.52E−01	4.64E−01
PTGR1 (GCST90087516)	MR Egger	1.11 (0.91–1.37)	4.14E−01		
	Weighted median	1.15 (1.03–1.29)	1.55E−02		
	Inverse variance weighted	1.15 (1.04–1.27)	6.65E−03	9.10E−01	7.53E−01

**Figure 2.**
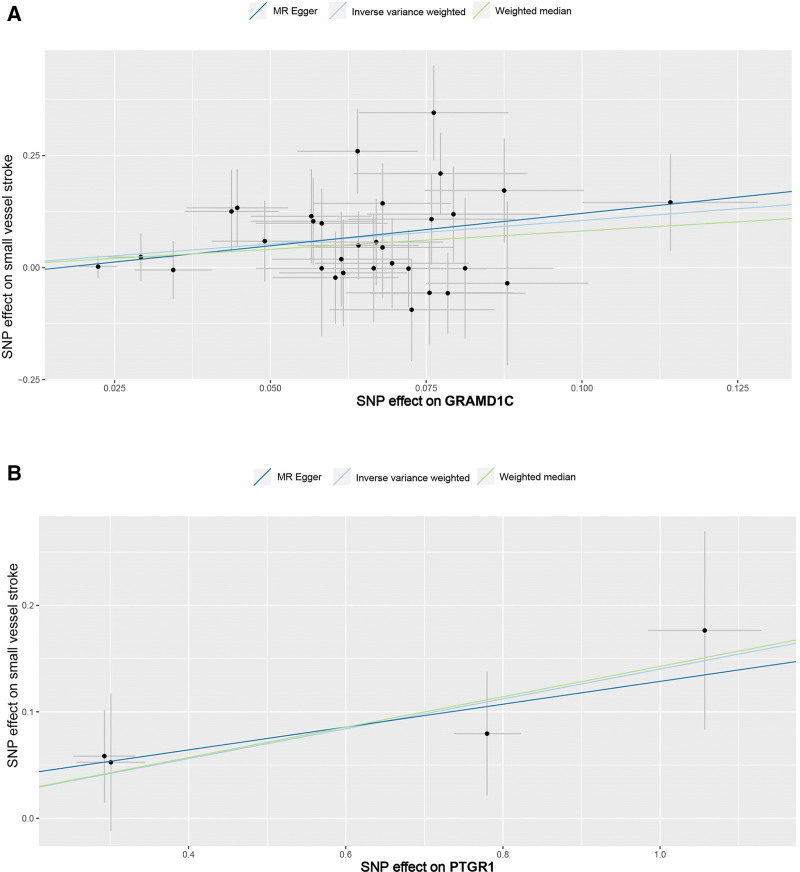
Scatter plot of (A) GRAMD1C and (B) PTGR1 on small vessel stroke. MR = Mendelian randomization.

**Figure 3.**
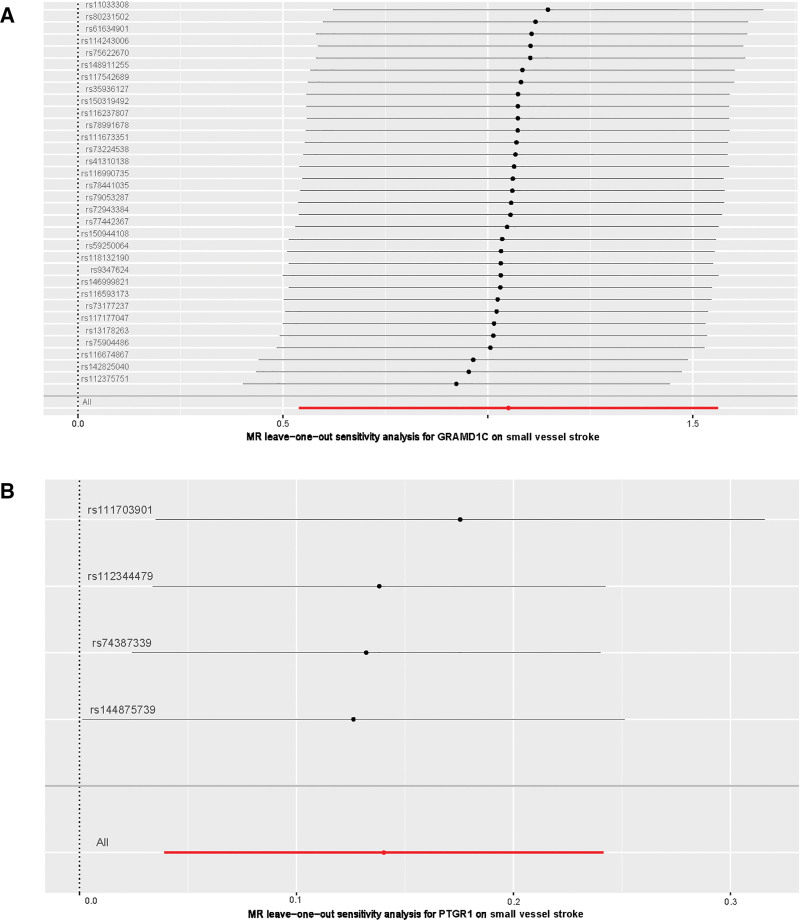
Leave-one-out plot of (A) GRAMD1C and (B) PTGR1 on small vessel stroke. MR = Mendelian randomization.

**Figure 4.**
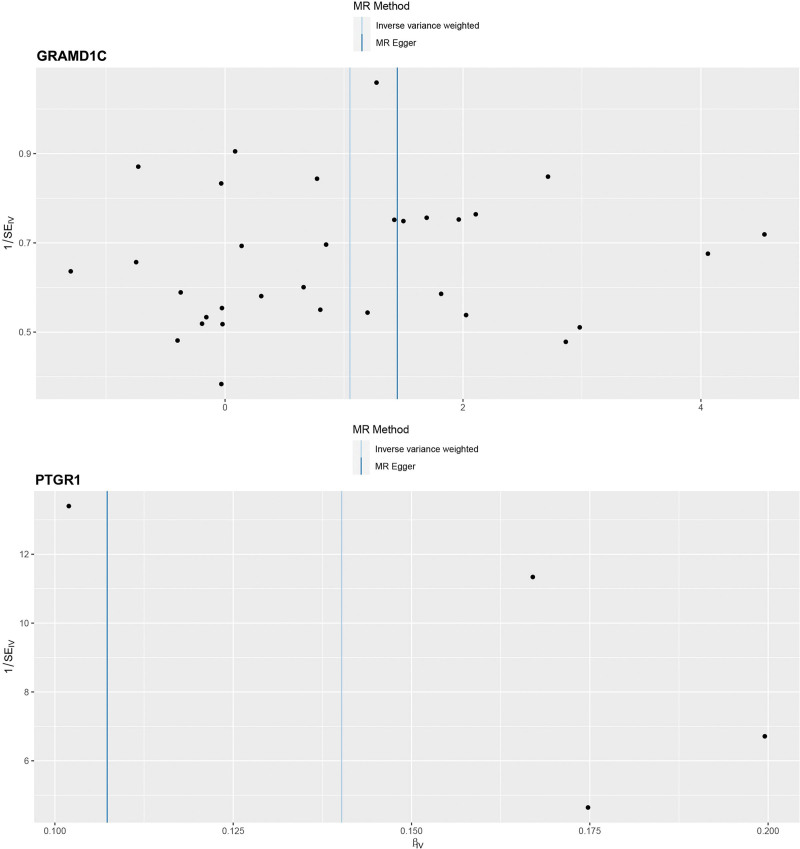
Funnel plot of GRAMD1C and PTGR1 on small vessel stroke. MR = Mendelian randomization.

For significant estimates of SVS, Cochran Q test (Table S1), MR-Egger intercept test (Table S1), scatter plots for MR analyses (Fig. 2), leave-one-out analysis (Fig. 3), and funnel plot (Fig. 4) were used to assess horizontal pleiotropy.

## 4. Discussion

This study explored the potential causal relationship between circulating proteins and SVS from a genetic perspective using a dual sample MR design method. We found GRAMD1C causally increased the risk of SVS (OR = 2.86, 95% CI = 1.71–4.76, *P* = 5.61e−05). In addition, this study suggested that PTGR1 was also associated with SVS.

GRAMD1C, a cholesterol transporter, is an evolutionarily conserved integral membrane protein found in the endoplasmic reticulum (ER), with most of the protein exposed to the cytosolic space.^[20]^ Through their research, Anne Simonsen team observed that the depletion or elimination of the GRAMD1C protein led to an increase in mitochondrial cholesterol content, a decrease in ER cholesterol content, and an improvement in mitochondrial ATP-coupled aerobic respiration rate. Interestingly, these effects were achieved without any impact on mitochondrial morphology, proteome, membrane potential, or cellular reactive oxygen species levels. These findings indicate that GRAMD1C protein may play a role in facilitating cholesterol transport from the ER to mitochondria, ultimately leading to the inhibition of mitochondrial bioenergy.^[21]^ Abnormal blood lipid metabolism, increase of circulating triglyceride and low-density lipoprotein cholesterol, and simultaneous decrease of high-density lipoprotein cholesterol promote cerebral atherosclerosis.^[22,23]^ There is a widespread belief that cerebral arteriosclerosis greatly increases the risk of SVS.^[24,25]^ Reports suggest that one strategy to prevent SVS is by increasing HDL-C.^[26]^ Despite the need for additional research to elucidate the specific mechanism, it is possible that GRAMD1C plays a role in promoting vascular sclerosis by mediating cholesterol transport, ultimately contributing to the occurrence of SVS.

In addition, this study also found that PTGR1 is related to the occurrence of SVS. PTGR1 is a prostaglandin reductase. Acting as a metabolic hub, PTGR1 plays a key role in inactivating the pro-inflammatory LTB4 and metabolizing the anti-inflammatory 17-oxo-DHA. This process helps in controlling the cellular levels of these crucial signaling lipids.^[27]^ Nevertheless, the precise pathogenic mechanism of PTGR1 on SVS remains unclear, necessitating further experimental research. However, Our discoveries may lead to new avenues of research and potential target proteins for researchers.

However, there are some limitations to the study. The exact mechanism by which GRAMD1C affects SVS is still unknown. While the MR method is useful for establishing causality, further confirmation through well-designed randomized controlled trials is necessary. The reliance on public GWAS data also presents challenges, as achieving complete non-overlap is difficult. In this study, the percentage of overlapping individuals is <1.30%.

## 5. Conclusion

This two-sample MR study found that a Cholesterol transporter (GRAMD1C) was causally associated with SVS and might constitute a promising target for diagnosis and therapy.

## Acknowledgments

We thank all the participants and investigators of proteomic GWAS derived from the NHGRI-EBI Catalog of human genome-wide association studies (https://www.ebi.ac.uk/gwas/). Four thousand, seven hundred eighty-two human circulating proteins were presented in Table S1. We thank the MEGASTROKE consortia for providing summary statistics data for stroke. The MEGASTROKE project received funding from sources specified at http://www.megastroke.org/acknowledgments.html. The investigator list of the MEGASTROKE consortium is presented in Table S1.

## Author contributions

**Conceptualization:** Ziwei Gao.

**Data curation:** Ziwei Gao, Peisen Yao.

**Formal analysis:** Yawen Xu, Yuanxiang Lin, Peisen Yao.

**Funding acquisition:** Yawen Xu, Dezhi Kang.

**Investigation:** Yawen Xu.

**Methodology:** Ziwei Gao, Peisen Yao.

**Project administration:** Yuanxiang Lin.

**Software:** Yawen Xu, Yue Chen, Peisen Yao.

**Supervision:** Yue Chen, Yuanxiang Lin.

**Validation:** Yue Chen, Dezhi Kang.

**Visualization:** Yawen Xu.

**Writing – original draft:** Ziwei Gao, Peisen Yao.

**Writing – review & editing:** Dezhi Kang, Yuanxiang Lin, Peisen Yao.




