# Supplementary material for: Circulating proteins and risk of small vessel stroke: A two-sample Mendelian randomization study
Source: Medicine (Baltimore). 2026 May 22;105(21):e48898. doi: 10.1097/MD.0000000000048898 (PMC13200920; doi:10.1097/MD.0000000000048898)
Supplement: Supplementary file 2 [file medi-105-e48898-s002.docx]

| **Table S2 Members of the MEGASTROKE CONSORTIUM** | |
| --- | --- |
| **Name** | **Institutional affiliations** |
| Rainer Malik | Institute for Stroke and Dementia Research (ISD), University Hospital, LMU Munich, Munich, Germany |
| Ganesh Chauhan | Centre for Brain Research, Indian Institute of Science, Bangalore, India |
| Matthew Traylor | Stroke Research Group, Division of Clinical Neurosciences, University of Cambridge, UK |
| Muralidharan Sargurupremraj | INSERM U1219 Bordeaux Population Health Research Center, Bordeaux, France |
|  | University of Bordeaux, Bordeaux, France |
| Yukinori Okada | Laboratory for Statistical Analysis, RIKEN Center for Integrative Medical Sciences, Yokohama, Japan |
|  | Department of Statistical Genetics, Osaka University Graduate School of Medicine, Osaka, Japan |
|  | Laboratory of Statistical Immunology, Immunology Frontier Research Center (WPI-IFReC), Osaka University, Suita, Japan. |
| Aniket Mishra | INSERM U1219 Bordeaux Population Health Research Center, Bordeaux, France |
|  | University of Bordeaux, Bordeaux, France |
| Loes Rutten-Jacobs | Stroke Research Group, Division of Clinical Neurosciences, University of Cambridge, UK |
| Anne-Katrin Giese | Department of Neurology, Massachusetts General Hospital, Harvard Medical School, Boston, MA, USA |
| Sander W van der Laan | Laboratory of Experimental Cardiology, Division of Heart and Lungs, University Medical Center Utrecht, University of Utrecht, Utrecht, Netherlands |
| Solveig Gretarsdottir | deCODE genetics/AMGEN inc, Reykjavik, Iceland |
| Christopher D Anderson | Center for Genomic Medicine, Massachusetts General Hospital (MGH), Boston, MA, USA |
|  | J. Philip Kistler Stroke Research Center, Department of Neurology, MGH, Boston, MA, USA |
|  | Program in Medical and Population Genetics, Broad Institute, Cambridge, MA, USA |
| Michael Chong | Population Health Research Institute, McMaster University, Hamilton, Canada |
| Hieab HH Adams | Department of Epidemiology, Erasmus University Medical Center, Rotterdam, Netherlands |
|  | Department of Radiology and Nuclear Medicine, Erasmus University Medical Center, Rotterdam, Netherlands |
| Tetsuro Ago | Department of Medicine and Clinical Science, Graduate School of Medical Sciences, Kyushu University, Fukuoka, Japan |
| Peter Almgren | Department of Clinical Sciences, Lund University, Malmö, Sweden |
| Philippe Amouyel | Univ. Lille, Inserm, Institut Pasteur de Lille, LabEx DISTALZ-UMR1167, Risk factors and molecular determinants of aging-related diseases, F-59000 Lille, France |
|  | Centre Hosp. Univ Lille, Epidemiology and Public Health Department, F-59000 Lille, France |
| Hakan Ay | AA Martinos Center for Biomedical Imaging, Department of Radiology, Massachusetts General Hospital, Harvard Medical School, Boston, MA, USA |
|  | J. Philip Kistler Stroke Research Center, Department of Neurology, MGH, Boston, MA, USA |
| Traci M Bartz | Cardiovascular Health Research Unit, Departments of Biostatistics and Medicine, University of Washington, Seattle, WA, USA |
| Oscar R Benavente | Division of Neurology, Faculty of Medicine, Brain Research Center, University of British Columbia, Vancouver, Canada |
| Steve Bevan | School of Life Science, University of Lincoln, Lincoln, UK |
| Giorgio B Boncoraglio | Department of Cerebrovascular Diseases, Fondazione IRCCS Istituto Neurologico "Carlo Besta", Milano, Italy |
| Robert D Brown, Jr. | Department of Neurology, Mayo Clinic Rochester, Rochester, MN, USA |
| Adam S Butterworth | MRC/BHF Cardiovascular Epidemiology Unit, Department of Public Health and Primary Care, University of Cambridge, Cambridge, UK |
|  | The National Institute for Health Research Blood and Transplant Research Unit in Donor Health and Genomics, University of Cambridge, UK |
| Caty Carrera | Neurovascular Research Laboratory, Vall d'Hebron Institut of Research, Neurology and Medicine Departments-Universitat Autònoma de Barcelona, Vall d’Hebrón Hospital, Barcelona, Spain |
|  | Stroke Pharmacogenomics and Genetics, Fundacio Docència i Recerca MutuaTerrassa, Terrassa, Spain |
| Cara L Carty | Children's Research Institute, Children's National Medical Center, Washington, DC, USA |
|  | Center for Translational Science, George Washington University, Washington, DC, USA |
| Daniel I Chasman | Division of Preventive Medicine, Brigham and Women's Hospital, Boston, MA, USA |
|  | Harvard Medical School, Boston, MA, USA |
| Wei-Min Chen | Center for Public Health Genomics, Department of Public Health Sciences, University of Virginia, Charlottesville, VA, USA |
| John W Cole | Department of Neurology, University of Maryland School of Medicine and Baltimore VAMC, Baltimore, MD, USA |
| Adolfo Correa | Departments of Medicine, Pediatrics and Population Health Science, University of Mississippi Medical Center, Jackson, MS, USA |
| Ioana Cotlarciuc | Institute of Cardiovascular Research, Royal Holloway University of London, UK & Ashford and St Peters Hospital, Surrey UK |
| Carlos Cruchaga | Department of Psychiatry, The Hope Center Program on Protein Aggregation and Neurodegeneration (HPAN), Washington University, School of Medicine, St. Louis, MO, USA |
|  | Department of Developmental Biology, Washington University School of Medicine, St. Louis, MO, USA |
| John Danesh | MRC/BHF Cardiovascular Epidemiology Unit, Department of Public Health and Primary Care, University of Cambridge, Cambridge, UK |
|  | NIHR Blood and Transplant Research Unit in Donor Health and Genomics, Department of Public Health and Primary Care, University of Cambridge, Cambridge, UK |
|  | Wellcome Trust Sanger Institute, Wellcome Trust Genome Campus, Hinxton, Cambridge, UK |
|  | British Heart Foundation, Cambridge Centre of Excellence, Department of Medicine, University of Cambridge, Cambridge, UK |
| Paul IW de Bakker | Department of Medical Genetics, University Medical Center Utrecht, Utrecht, Netherlands |
|  | Department of Epidemiology, Julius Center for Health Sciences and Primary Care, University Medical Center Utrecht, Utrecht, Netherlands |
| Marcel den Hoed | Department of Immunology, Genetics and Pathology and Science for Life Laboratory, Uppsala University, Uppsala, Sweden |
| Qing Duan | Department of Genetics, University of North Carolina, Chapel Hill, NC, USA |
| Stefan T Engelter | Department of Neurology and Stroke Center, Basel University Hospital, Switzerland |
|  | Neurorehabilitation Unit, University and University Center for Medicine of Aging and Rehabilitation Basel, Felix Platter Hospital, Basel, Switzerland |
| Guido J Falcone | Department of Neurology, Yale University School of Medicine, New Haven, CT, USA |
|  | Program in Medical and Population Genetics, The Broad Institute of Harvard and MIT, Cambridge, MA, USA |
| Rebecca F Gottesman | Department of Neurology, Johns Hopkins University School of Medicine, Baltimore, MD, USA |
| Anita L DeStefano | Boston University School of Public Health, Boston, MA, USA |
|  | Framingham Heart Study, Framingham, MA, USA |
| Raji P Grewal | Neuroscience Institute, SF Medical Center, Trenton, NJ, USA |
| Vilmundur Gudnason | Icelandic Heart Association Research Institute, Kopavogur, Iceland |
|  | University of Iceland, Faculty of Medicine, Reykjavik, Iceland |
| Stefan Gustafsson | Department of Medical Sciences, Molecular Epidemiology and Science for Life Laboratory, Uppsala University, Uppsala, Sweden |
| Jeffrey Haessler | Division of Public Health Sciences, Fred Hutchinson Cancer Research Center, Seattle, WA, USA |
| Tamara B Harris | Laboratory of Epidemiology and Population Science, National Institute on Aging, National Institutes of Health, Bethesda, MD, USA |
| Ahamad Hassan | Department of Neurology, Leeds General Infirmary, Leeds Teaching Hospitals NHS Trust, Leeds, UK |
| Aki S Havulinna | National Institute for Health and Welfare, Helsinki, Finland |
|  | FIMM - Institute for Molecular Medicine Finland, Helsinki, Finland |
| Susan R Heckbert | Department of Epidemiology, University of Washington, Seattle, WA, USA |
| Elizabeth G Holliday | Public Health Stream, Hunter Medical Research Institute, New Lambton, Australia |
|  | Faculty of Health and Medicine, University of Newcastle, Newcastle, Australia |
| George Howard | School of Public Health, University of Alabama at Birmingham, Birmingham, AL, USA |
| Fang-Chi Hsu | Department of Biostatistical Sciences, Wake Forest School of Medicine, Winston-Salem, NC, USA |
| Hyacinth I Hyacinth | Aflac Cancer and Blood Disorder Center, Department of Pediatrics, Emory University School of Medicine, Atlanta, GA, USA |
| M Arfan Ikram | Department of Epidemiology, Erasmus University Medical Center, Rotterdam, Netherlands |
| Erik Ingelsson | Department of Medicine, Division of Cardiovascular Medicine, Stanford University School of Medicine, CA, USA |
|  | Department of Medical Sciences, Molecular Epidemiology and Science for Life Laboratory, Uppsala University, Uppsala, Sweden |
| Marguerite R Irvin | Epidemiology, School of Public Health, University of Alabama at Birmingham, USA |
| Xueqiu Jian | Brown Foundation Institute of Molecular Medicine, University of Texas Health Science Center at Houston, Houston, TX, USA |
| Jordi Jiménez-Conde | Neurovascular Research Group (NEUVAS), Neurology Department, Institut Hospital del Mar d'Investigació Mèdica, Universitat Autònoma de Barcelona, Barcelona, Spain |
| Julie A Johnson | Department of Pharmacotherapy and Translational Research and Center for Pharmacogenomics, University of Florida, College of Pharmacy, Gainesville, FL, USA |
|  | Division of Cardiovascular Medicine, College of Medicine, University of Florida, Gainesville, FL, USA |
| J Wouter Jukema | Department of Cardiology, Leiden University Medical Center, Leiden, the Netherlands |
| Masahiro Kanai | Laboratory for Statistical Analysis, RIKEN Center for Integrative Medical Sciences, Yokohama, JapanDepartment of Statistical Genetics, Osaka University Graduate School of Medicine, Osaka, Japan |
|  | Program in Bioinformatics and Integrative Genomics, Harvard Medical School, Boston, MA, USA |
| Keith L Keene | Department of Biology, East Carolina University, Greenville, NC, USA |
|  | Center for Health Disparities, East Carolina University, Greenville, NC, USA |
| Brett M Kissela | University of Cincinnati College of Medicine, Cincinnati, OH, USA |
| Dawn O Kleindorfer | University of Cincinnati College of Medicine, Cincinnati, OH, USA |
| Charles Kooperberg | Division of Public Health Sciences, Fred Hutchinson Cancer Research Center, Seattle, WA, USA |
| Michiaki Kubo | RIKEN Center for Integrative Medical Sciences, Yokohama, Japan |
| Leslie A Lange | Department of Medicine, University of Colorado Denver, Anschutz Medical Campus, Aurora, CO, USA |
| Carl D Langefeld | Center for Public Health Genomics and Department of Biostatistical Sciences, Wake Forest School of Medicine, Winston-Salem, NC, USA |
| Claudia Langenberg | MRC Epidemiology Unit, University of Cambridge School of Clinical Medicine, Institute of Metabolic Science, Cambridge Biomedical Campus, Cambridge, UK |
| Lenore J Launer | Intramural Research Program, National Institute on Aging, National Institutes of Health, Bethesda, MD, USA |
| Jin-Moo Lee | Department of Neurology, Radiology, and Biomedical Engineering, Washington University School of Medicine, St. Louis, MO, USA |
| Robin Lemmens | KU Leuven – University of Leuven, Department of Neurosciences, Experimental Neurology, Leuven, Belgium |
|  | VIB Center for Brain & Disease Research, University Hospitals Leuven, Department of Neurology, Leuven, Belgium |
| Didier Leys | Univ.-Lille, INSERM U 1171. CHU Lille. Lille, France |
| Cathryn M Lewis | Department of Medical and Molecular Genetics, King's College London, London, UK |
|  | SGDP Centre, Institute of Psychiatry, Psychology & Neuroscience, King's College London, London, UK |
| Wei-Yu Lin | MRC/BHF Cardiovascular Epidemiology Unit, Department of Public Health and Primary Care, University of Cambridge, Cambridge, UK |
|  | Northern Institute for Cancer Research, Paul O'Gorman Building, Newcastle University, Newcastle, UK |
| Arne G Lindgren | Department of Clinical Sciences Lund, Neurology, Lund University, Lund, Sweden |
|  | Department of Neurology and Rehabilitation Medicine, Skåne University Hospital, Lund, Sweden |
| Erik Lorentzen | Bioinformatics Core Facility, University of Gothenburg, Gothenburg, Sweden |
| Patrik K Magnusson | Department of Medical Epidemiology and Biostatistics, Karolinska Institutet, Stockholm, Sweden |
| Jane Maguire | University of Technology Sydney, Faculty of Health, Ultimo, Australia |
| Ani Manichaikul | Center for Public Health Genomics, Department of Public Health Sciences, University of Virginia, Charlottesville, VA, USA |
| Patrick F McArdle | Department of Medicine, University of Maryland School of Medicine,MD, USA |
| James F Meschia | Department of Neurology, Mayo Clinic, Jacksonville, FL, USA |
| Braxton D Mitchell | Department of Medicine, University of Maryland School of Medicine,MD, USA |
|  | Geriatrics Research and Education Clinical Center, Baltimore Veterans Administration Medical Center, Baltimore, MD, USA |
| Thomas H Mosley | Division of Geriatrics, School of Medicine, University of Mississippi Medical Center, Jackson, MS, USA |
|  | Memory Impairment and Neurodegenerative Dementia Center, University of Mississippi Medical Center, Jackson, MS, USA |
| Michael A Nalls | Laboratory of Neurogenetics, National Institute on Aging, National institutes of Health, Bethesda, MD, USA |
|  | Data Tecnica International, Glen Echo MD, USA |
| Toshiharu Ninomiya | Department of Epidemiology and Public Health, Graduate School of Medical Sciences, Kyushu University, Fukuoka, Japan |
| Martin J O'Donnell | Population Health Research Institute, McMaster University, Hamilton, Canada |
|  | Clinical Research Facility, Department of Medicine, NUI Galway, Galway, Ireland |
| Bruce M Psaty | Cardiovascular Health Research Unit, Department of Medicine, University of Washington, Seattle, WA, USA |
|  | Department of Epidemiology, University of Washington, Seattle, WA |
|  | Department of Health Services, University of Washington, Seattle, WA, USA |
|  | Kaiser Permanente Washington Health Research Institute, Seattle, WA, USA |
| Sara L Pulit | Brain Center Rudolf Magnus, Department of Neurology, University Medical Center Utrecht, Utrecht, The Netherlands |
|  | Department of Medical Genetics, University Medical Center Utrecht, Utrecht, Netherlands |
| Kristiina Rannikmäe | Usher Institute of Population Health Sciences and Informatics, University of Edinburgh, Edinburgh, UK |
|  | Centre for Clinical Brain Sciences, University of Edinburgh, Edinburgh, UK |
| Alexander P Reiner | Department of Epidemiology, University of Washington, Seattle, WA, USA |
|  | Fred Hutchinson Cancer Research Center, University of Washington, Seattle, WA, USA |
| Kathryn M Rexrode | Department of Medicine, Brigham and Women's Hospital, Boston, MA, USA |
| Kenneth Rice | Department of Biostatistics, University of Washington, Seattle, WA, USA |
| Stephen S Rich | Center for Public Health Genomics, Department of Public Health Sciences, University of Virginia, Charlottesville, VA, USA |
| Paul M Ridker | Division of Preventive Medicine, Brigham and Women's Hospital, Boston, USA |
|  | Harvard Medical School, Boston, MA, USA |
| Natalia S Rost | Department of Neurology, Massachusetts General Hospital, Harvard Medical School, Boston, MA, USA |
|  | J. Philip Kistler Stroke Research Center, Department of Neurology, MGH, Boston, MA, USA |
| Peter M Rothwell | Nuffield Department of Clinical Neurosciences, University of Oxford, UK |
| Jerome I Rotter | Institute for Translational Genomics and Population Sciences, Los Angeles Biomedical Research Institute at Harbor-UCLA Medical Center, Torrance, CA, USA |
|  | Division of Genomic Outcomes, Department of Pediatrics, Harbor-UCLA Medical Center, Torrance, CA, USA |
| Tatjana Rundek | Department of Neurology, Miller School of Medicine, University of Miami, Miami, FL, USA |
| Ralph L Sacco | Department of Neurology, Miller School of Medicine, University of Miami, Miami, FL, USA |
| Saori Sakaue | Department of Statistical Genetics, Osaka University Graduate School of Medicine, Osaka, Japan |
|  | Department of Allergy and Rheumatology, Graduate School of Medicine, the University of Tokyo, Tokyo, Japan |
| Michele M Sale | Center for Public Health Genomics, University of Virginia, Charlottesville, VA, USA |
| Veikko Salomaa | National Institute for Health and Welfare, Helsinki, Finland |
| Bishwa R Sapkota | Department of Pediatrics, College of Medicine, University of Oklahoma Health Sciences Center, Oklahoma City, OK, USA |
| Reinhold Schmidt | Department of Neurology, Medical University of Graz, Graz, Austria |
| Carsten O Schmidt | University Medicine Greifswald, Institute for Community Medicine, SHIP-KEF, Greifswald, Germany |
| Ulf Schminke | University Medicine Greifswald, Department of Neurology, Greifswald, Germany |
| Pankaj Sharma | Institute of Cardiovascular Research, Royal Holloway University of London, UK & Ashford and St Peters Hospital, Surrey UK |
| Agnieszka Slowik | Department of Neurology, Jagiellonian University, Krakow, Poland |
| Cathie LM Sudlow | Usher Institute of Population Health Sciences and Informatics, University of Edinburgh, Edinburgh, UK |
|  | Centre for Clinical Brain Sciences, University of Edinburgh, Edinburgh, UK |
| Christian Tanislav | Department of Neurology, Justus Liebig University, Giessen, Germany |
| Turgut Tatlisumak | Department of Clinical Neurosciences/Neurology, Institute of Neuroscience and Physiology, Sahlgrenska Academy at University of Gothenburg, Gothenburg, Sweden |
|  | Sahlgrenska University Hospital, Gothenburg, Sweden |
| Kent D Taylor | Institute for Translational Genomics and Population Sciences, Los Angeles Biomedical Research Institute at Harbor-UCLA Medical Center, Torrance, USA |
|  | Division of Genomic Outcomes, Department of Pediatrics, Harbor-UCLA Medical Center, Torrance, CA, USA |
| Vincent NS Thijs | Stroke Division, Florey Institute of Neuroscience and Mental Health, University of Melbourne, Heidelberg, Australia |
|  | Austin Health, Department of Neurology, Heidelberg, Australia |
| Gudmar Thorleifsson | deCODE genetics/AMGEN inc, Reykjavik, Iceland |
| Unnur Thorsteinsdottir | deCODE genetics/AMGEN inc, Reykjavik, Iceland |
| Steffen Tiedt | Institute for Stroke and Dementia Research (ISD), University Hospital, LMU Munich, Munich, Germany |
| Stella Trompet | Department of Internal Medicine, Section Gerontology and Geriatrics, Leiden University Medical Center, Leiden, the Netherlands |
| Christophe Tzourio | University of Bordeaux, Bordeaux, France |
|  | INSERM U1219, Bordeaux, France |
|  | Department of Public Health, Bordeaux University Hospital, Bordeaux, France |
| Cornelia M van Duijn | Genetic Epidemiology Unit, Department of Epidemiology, Erasmus University Medical Center Rotterdam, Netherlands |
|  | Center for Medical Systems Biology, Leiden, Netherlands |
| Matthew Walters | School of Medicine, Dentistry and Nursing at the University of Glasgow, Glasgow, UK |
| Nicholas J Wareham | MRC Epidemiology Unit, University of Cambridge School of Clinical Medicine, Institute of Metabolic Science, Cambridge Biomedical Campus, UK |
| Sylvia Wassertheil-Smoller | Department of Epidemiology and Population Health, Albert Einstein of Medicine, NY, USA |
| James G Wilson | Department of Physiology and Biophysics, University of Mississippi Medical Center, Jackson, MS, USA |
| Kerri L Wiggins | Cardiovascular Health Research Unit, Department of Medicine, University of Washington, Seattle, WA, USA |
| Qiong Yang | Boston University School of Public Health, Boston, MA, USA |
| Salim Yusuf | Population Health Research Institute, McMaster University, Hamilton, Canada |
| Najaf Amin | Department of Epidemiology, Erasmus University Medical Center, Rotterdam, Netherlands |
| Hugo S Aparicio | Boston University School of Medicine, Boston, MA, USA |
|  | Framingham Heart Study, Framingham, MA, USA |
| Donna K Arnett | University of Kentucky College of Public Health, Lexington, KY, USA |
|  | A full list of members and affiliations appears in the Supplementary Note |
|  | Department of Human Genetics, McGill University, Montreal, Canada |
| John Attia | University of Newcastle and Hunter Medical Research Institute, New Lambton, Australia |
| Alexa S Beiser | Boston University School of Public Health, Boston, MA, USA |
|  | Framingham Heart Study, Framingham, MA, USA |
| Claudine Berr | Univ. Montpellier, Inserm, U1061, Montpellier, France |
| Julie E Buring | Division of Preventive Medicine, Brigham and Women's Hospital, Boston, USA |
|  | Harvard Medical School, Boston, MA, USA |
| Mariana Bustamante | Centre for Research in Environmental Epidemiology, Barcelona, Spain |
| Valeria Caso | Department of Neurology, Università degli Studi di Perugia, Umbria, Italy |
| Yu-Ching Cheng | Department of Medicine, University of Maryland School of Medicine, Baltimore, MD, USA |
| Seung Hoan Choi | Broad Institute, Cambridge, MA, USA |
|  | Framingham Heart Study, Framingham, MA, USA |
| Ayesha Chowhan | Boston University School of Medicine, Boston, MA, USA |
|  | Framingham Heart Study, Framingham, MA, USA |
| Natalia Cullell | Stroke Pharmacogenomics and Genetics, Fundacio Docència i Recerca MutuaTerrassa, Terrassa, Spain |
| Jean-François Dartigues | Univ. Bordeaux, Inserm, Bordeaux Population Health Research Center, UMR 1219, Bordeaux, France |
|  | Bordeaux University Hospital, Department of Neurology, Memory Clinic, Bordeaux, France |
| Hossein Delavaran | Department of Clinical Sciences Lund, Neurology, Lund University, Lund, Sweden |
|  | Department of Neurology and Rehabilitation Medicine, Skåne University Hospital, Lund, Sweden |
| Pilar Delgado | Neurovascular Research Laboratory. Vall d'Hebron Institut of Research, Neurology and Medicine Departments-Universitat Autònoma de Barcelona. Vall d’Hebrón Hospital, Barcelona, Spain |
| Marcus Dörr | University Medicine Greifswald, Department of Internal Medicine B, Greifswald, Germany |
|  | DZHK, Greifswald, Germany |
| Gunnar Engström | Department of Clinical Sciences, Lund University, Malmö, Sweden |
| Ian Ford | Robertson Center for Biostatistics, University of Glasgow, Glasgow, UK |
| Wander S Gurpreet | Hero DMC Heart Institute, Dayanand Medical College & Hospital, Ludhiana, India |
| Anders Hamsten | Atherosclerosis Research Unit, Department of Medicine Solna, Karolinska Institutet, Stockholm, Sweden |
|  | Karolinska Institutet, Stockholm, Sweden |
| Laura Heitsch | Division of Emergency Medicine, and Department of Neurology, Washington University School of Medicine, St. Louis, MO, USA |
| Atsushi Hozawa | Tohoku Medical Megabank Organization, Sendai, Japan |
| Laura Ibanez | Department of Psychiatry, Washington University School of Medicine, St. Louis, MO, USA |
| Andreea Ilinca | Department of Clinical Sciences Lund, Neurology, Lund University, Lund, Sweden |
|  | Department of Neurology and Rehabilitation Medicine, Skåne University Hospital, Lund, Sweden |
| Martin Ingelsson | Department of Public Health and Caring Sciences / Geriatrics, Uppsala University, Uppsala, Sweden |
| Motoki Iwasaki | Epidemiology and Prevention Group, Center for Public Health Sciences, National Cancer Center, Tokyo, Japan |
| Rebecca D Jackson | Department of Internal Medicine and the Center for Clinical and Translational Science, The Ohio State University, Columbus, OH, USA |
| Katarina Jood | Institute of Neuroscience and Physiology, the Sahlgrenska Academy at University of Gothenburg, Gothenburg, Sweden |
| Pekka Jousilahti | National Institute for Health and Welfare, Helsinki, Finland |
| Sara Kaffashian | INSERM U1219 Bordeaux Population Health Research Center, Bordeaux, France |
|  | University of Bordeaux, Bordeaux, France |
| Lalit Kalra | Department of Basic and Clinical Neurosciences, King's College London, London, UK |
| Masahiro Kamouchi | Department of Health Care Administration and Management, Graduate School of Medical Sciences, Kyushu University, Japan |
| Takanari Kitazono | Department of Medicine and Clinical Science, Graduate School of Medical Sciences, Kyushu University, Japan |
| Olafur Kjartansson | Landspitali National University Hospital, Departments of Neurology & Radiology, Reykjavik, Iceland |
| Manja Kloss | Department of Neurology, Heidelberg University Hospital, Germany |
| Peter J Koudstaal | Department of Neurology, Erasmus University Medical Center |
| Jerzy Krupinski | Hospital Universitari Mutua Terrassa, Terrassa (Barcelona), Spain |
| Daniel L Labovitz | Albert Einstein College of Medicine, Montefiore Medical Center, New York, USA |
| Cathy C Laurie | Department of Biostatistics, University of Washington, Seattle, WA, USA |
| Christopher R Levi | John Hunter Hospital, Hunter Medical Research Institute and University of Newcastle, Newcastle, NSW, Australia |
| Linxin Li | Centre for Prevention of Stroke and Dementia, Nuffield Department of Clinical Neurosciences, University of Oxford, UK |
| Lars Lind | Department of Medical Sciences, Uppsala University, Uppsala, Sweden |
| Cecilia M Lindgren | Genetic and Genomic Epidemiology Unit, Wellcome Trust Centre for Human Genetics, University of Oxford, Oxford, UK |
|  | The Wellcome Trust Centre for Human Genetics, Oxford, UK |
| Vasileios Lioutas | Beth Israel Deaconess Medical Center, Boston, MA, USA |
|  | Framingham Heart Study, Framingham, MA, USA |
| Yong Mei Liu | Wake Forest School of Medicine, Wake Forest, NC, USA |
| Oscar L Lopez | Department of Neurology, University of Pittsburgh, Pittsburgh, PA, USA |
| Hirata Makoto | BioBank Japan, Laboratory of Clinical Sequencing, Department of Computational biology and medical Sciences, Graduate school of Frontier Sciences, The University of Tokyo, Tokyo, Japan |
| Nicolas Martinez-Majander | Department of Neurology, Helsinki University Hospital, Helsinki, Finland |
| Koichi Matsuda | BioBank Japan, Laboratory of Clinical Sequencing, Department of Computational biology and medical Sciences, Graduate school of Frontier Sciences, The University of Tokyo, Tokyo, Japan |
| Naoko Minegishi | Tohoku Medical Megabank Organization, Sendai, Japan |
| Joan Montaner | Neurovascular Research Laboratory, Vall d'Hebron Institut of Research, Neurology and Medicine Departments-Universitat Autònoma de Barcelona. Vall d’Hebrón Hospital, Barcelona, Spain |
| Andrew P Morris | Department of Biostatistics, University of Liverpool, Liverpool, UK |
|  | Wellcome Trust Centre for Human Genetics, University of Oxford, Oxford, UK |
| Elena Muiño | Stroke Pharmacogenomics and Genetics, Fundacio Docència i Recerca MutuaTerrassa, Terrassa, Spain |
| Martina Müller-Nurasyid | Institute of Genetic Epidemiology, Helmholtz Zentrum München - German Research Center for Environmental Health, Neuherberg, Germany |
|  | Department of Medicine I, Ludwig-Maximilians-Universität, Munich, Germany |
|  | DZHK (German Centre for Cardiovascular Research), partner site Munich Heart Alliance, Munich, Germany |
| Bo Norrving | Department of Clinical Sciences Lund, Neurology, Lund University, Lund, Sweden |
|  | Department of Neurology and Rehabilitation Medicine, Skåne University Hospital, Lund, Sweden |
| Soichi Ogishima | Tohoku Medical Megabank Organization, Sendai, Japan |
| Eugenio A Parati | Department of Cerebrovascular Diseases, Fondazione IRCCS Istituto Neurologico “Carlo Besta”, Milano, Italy |
| Leema Reddy Peddareddygari | Neuroscience Institute, SF Medical Center, Trenton, NJ, USA |
| Nancy L Pedersen | Department of Medical Epidemiology and Biostatistics, Karolinska Institutet, Stockholm, Sweden |
| Joanna Pera | Department of Neurology, Jagiellonian University, Krakow, Poland |
| Markus Perola | National Institute for Health and Welfare, Helsinki, Finland |
|  | University of Tartu, Estonian Genome Center, Tartu, Estonia, Tartu, Estonia |
| Alessandro Pezzini | Department of Clinical and Experimental Sciences, Neurology Clinic, University of Brescia, Italy |
| Silvana Pileggi | Translational Genomics Unit, Department of Oncology, IRCCS Istituto di Ricerche Farmacologiche Mario Negri, Milano, Italy |
| Raquel Rabionet | Department of Genetics, Microbiology and Statistics, University of Barcelona, Barcelona, Spain |
| Iolanda Riba-Llena | Neurovascular Research Laboratory, Vall d'Hebron Institut of Research, Neurology and Medicine Departments-Universitat Autònoma de Barcelona, Vall d’Hebrón Hospital, Barcelona, Spain |
| Marta Ribasés | Psychiatric Genetics Unit, Group of Psychiatry, Mental Health and Addictions, Vall d’Hebron Research Institute (VHIR), Universitat Autònoma de Barcelona, Biomedical Network Research Centre on Mental Health (CIBERSAM), Barcelona, Spain |
| Jose R Romero | Boston University School of Medicine, Boston, MA, USA |
|  | Framingham Heart Study, Framingham, MA, USA |
| Jaume Roquer | Department of Neurology, IMIM-Hospital del Mar, and Universitat Autònoma de Barcelona, Spain |
|  | IMIM (Hospital del Mar Medical Research Institute), Barcelona, Spain |
| Anthony G Rudd | National Institute for Health Research Comprehensive Biomedical Research Centre, Guy's & St. Thomas' NHS Foundation Trust and King's College London, London, UK |
|  | Division of Health and Social Care Research, King's College London, UK |
| Antti-Pekka Sarin | FIMM-Institute for Molecular Medicine Finland, Helsinki, Finland |
|  | THL-National Institute for Health and Welfare, Helsinki, Finland |
| Ralhan Sarju | Hero DMC Heart Institute, Dayanand Medical College & Hospital, Ludhiana, India |
| Chloe Sarnowski | Boston University School of Public Health, Boston, MA, USA |
|  | Framingham Heart Study, Framingham, MA, USA |
| Makoto Sasaki | Iwate Tohoku Medical Megabank Organization, Iwate Medical University, Iwate, Japan |
| Claudia L Satizabal | Boston University School of Medicine, Boston, MA, USA |
|  | Framingham Heart Study, Framingham, MA, USA |
| Mamoru Satoh | Iwate Tohoku Medical Megabank Organization, Iwate Medical University, Iwate, Japan |
| Naveed Sattar | BHF Glasgow Cardiovascular Research Centre, Faculty of Medicine, Glasgow, UK |
| Norie Sawada | Epidemiology and Prevention Group, Center for Public Health Sciences, National Cancer Center, Tokyo, Japan |
| Gerli Sibolt | Department of Neurology, Helsinki University Hospital, Helsinki, Finland |
| Ásgeir Sigurdsson | deCODE Genetics/Amgen, Inc., Reykjavik, Iceland |
| Albert Smith | Icelandic Heart Association, Reykjavik, Iceland |
| Kenji Sobue | Iwate Tohoku Medical Megabank Organization, Iwate Medical University, Iwate, Japan |
| Carolina Soriano-Tárraga | IMIM (Hospital del Mar Medical Research Institute), Barcelona, Spain |
| Tara Stanne | Institute of Biomedicine, the Sahlgrenska Academy at University of Gothenburg, Gothenburg, Sweden |
| Colin Stine | Department of Epidemiology, University of Maryland School of Medicine, Baltimore, MD, USA |
| David J Stott | Institute of Cardiovascular and Medical Sciences, Faculty of Medicine, University of Glasgow, Glasgow, UK |
| Konstantin Strauch | Institute of Genetic Epidemiology, Helmholtz Zentrum München - German Research Center for Environmental Health, Neuherberg, Germany |
|  | Chair of Genetic Epidemiology, IBE, Faculty of Medicine, LMU Munich, Germany |
| O Takako Takai | Tohoku Medical Megabank Organization, Sendai, Japan |
| Hideo Tanaka | Division of Epidemiology and Prevention, Aichi Cancer Center Research Institute, Nagoya, Japan |
|  | Department of Epidemiology, Nagoya University Graduate School of Medicine, Nagoya, Japan |
| Kozo Tanno | Iwate Tohoku Medical Megabank Organization, Iwate Medical University, Iwate, Japan |
| Alexander Teumer | University Medicine Greifswald, Institute for Community Medicine, SHIP-KEF, Greifswald, Germany |
| Liisa Tomppo | Clinical Neurosciences, Neurology, University of Helsinki, Helsinki, Finland |
| Nuria P Torres-Aguila | Stroke Pharmacogenomics and Genetics, Fundacio Docència i Recerca MutuaTerrassa, Terrassa, Spain |
| Emmanuel Touze | Department of Neurology, Caen University Hospital, Caen, France |
|  | University of Caen Normandy, Caen, France |
| Shoichiro Tsugane | Epidemiology and Prevention Group, Center for Public Health Sciences, National Cancer Center, Tokyo, Japan |
| Andre G Uitterlinden | Department of Internal Medicine, Erasmus University Medical Center, Rotterdam, Netherlands |
| Einar M Valdimarsson | Landspitali University Hospital, Reykjavik, Iceland |
| Sven J van der Lee | Department of Epidemiology, Erasmus University Medical Center, Rotterdam, Netherlands |
| Henry Völzke | University Medicine Greifswald, Institute for Community Medicine, SHIP-KEF, Greifswald, Germany |
| Kenji Wakai | Division of Epidemiology and Prevention, Aichi Cancer Center Research Institute, Nagoya, Japan |
| David Weir | Survey Research Center, University of Michigan, Ann Arbor, MI, USA |
| Stephen R Williams | University of Virginia Department of Neurology, Charlottesville, VA, USA |
| Charles DA Wolfe | National Institute for Health Research Comprehensive Biomedical Research Centre, Guy's & St. Thomas' NHS Foundation Trust and King's College London, London, UK |
|  | Division of Health and Social Care Research, King's College London, London, UK |
| Quenna Wong | Department of Biostatistics, University of Washington, Seattle, WA, USA |
| Huichun Xu | Department of Medicine, University of Maryland School of Medicine, Baltimore, MD, USA |
| Taiki Yamaji | Epidemiology and Prevention Group, Center for Public Health Sciences, National Cancer Center, Tokyo, Japan |
| Dharambir K Sanghera | Department of Pediatrics, College of Medicine, University of Oklahoma Health Sciences Center, Oklahoma City, OK, USA |
|  | Department of Phamaceutical Sciences, Collge of Pharmacy, University of Oklahoma Health Sciences Center, Oklahoma City, OK, USA |
|  | Oklahoma Center for Neuroscience, Oklahoma City, OK, USA |
| Olle Melander | Department of Clinical Sciences, Lund University, Malmö, Sweden |
| Christina Jern | Department of Pathology and Genetics, Institute of Biomedicine, The Sahlgrenska Academy at University of Gothenburg, Gothenburg, Sweden |
| Daniel Strbian | Department of Neurology, Helsinki University Hospital, Helsinki, Finland |
|  | Clinical Neurosciences, Neurology, University of Helsinki, Helsinki, Finland |
| Israel Fernandez-Cadenas | Stroke Pharmacogenomics and Genetics, Fundacio Docència i Recerca MutuaTerrassa, Terrassa, Spain |
|  | Neurovascular Research Laboratory, Vall d'Hebron Institut of Research, Neurology and Medicine Departments-Universitat Autònoma de Barcelona, Vall d’Hebrón Hospital, Barcelona, Spain |
| W T Longstreth, Jr | Department of Neurology, University of Washington, Seattle, WA, USA |
|  | Department of Epidemiology, University of Washington, Seattle, WA, USA |
| Arndt Rolfs | Albrecht Kossel Institute, University Clinic of Rostock, Rostock, Germany |
| Jun Hata | Department of Epidemiology and Public Health, Graduate School of Medical Sciences, Kyushu University, Fukuoka, Japan |
| Daniel Woo | University of Cincinnati College of Medicine, Cincinnati, OH, USA |
| Jonathan Rosand | Center for Genomic Medicine, Massachusetts General Hospital (MGH), Boston, MA, USA |
|  | J. Philip Kistler Stroke Research Center, Department of Neurology, MGH, Boston, MA, USA |
|  | Program in Medical and Population Genetics, Broad Institute, Cambridge, MA, USA |
| Guillaume Pare | Population Health Research Institute, McMaster University, Hamilton, Canada |
| Jemma C Hopewell | Clinical Trial Service Unit and Epidemiological Studies Unit, Nuffield Department of Population Health, University of Oxford, Oxford, UK |
| Danish Saleheen | Department of Genetics, Perelman School of Medicine, University of Pennsylvania, PA, USA |
| Kari Stefansson | deCODE genetics/AMGEN inc, Reykjavik, Iceland |
|  | Faculty of Medicine, University of Iceland, Reykjavik, Iceland |
| Bradford B Worrall | Departments of Neurology and Public Health Sciences, University of Virginia School of Medicine, Charlottesville, VA, USA |
| Steven J Kittner | Department of Neurology, University of Maryland School of Medicine and Baltimore VAMC, Baltimore, MD, USA |
| Sudha Seshadri | Department of Neurology, Boston University School of Medicine, Boston, MA, USA |
|  | Framingham Heart Study, Framingham, MA, USA |
| Myriam Fornage | Brown Foundation Institute of Molecular Medicine, University of Texas Health Science Center at Houston, Houston, TX, USA |
|  | Human Genetics Center, University of Texas Health Science Center at Houston, Houston, TX, USA |
| Hugh S Markus | Stroke Research Group, Division of Clinical Neurosciences, University of Cambridge, UK |
| Joanna MM Howson | MRC/BHF Cardiovascular Epidemiology Unit, Department of Public Health and Primary Care, University of Cambridge, Cambridge, UK |
| Yoichiro Kamatani | Laboratory for Statistical Analysis, RIKEN Center for Integrative Medical Sciences, Yokohama, Japan |
|  | Center for Genomic Medicine, Kyoto University Graduate School of Medicine, Kyoto, Japan |
| Stephanie Debette | INSERM U1219 Bordeaux Population Health Research Center, Bordeaux, France |
|  | University of Bordeaux, Bordeaux, France |
| Martin Dichgans | Institute for Stroke and Dementia Research (ISD), University Hospital, LMU Munich, Munich, Germany |
|  | Munich Cluster for Systems Neurology (SyNergy), Munich, Germany |
|  | German Center for Neurodegenerative Diseases (DZNE), Munich, Germany |
